# Depression and Replacement of the Superior Maxilla (Langenbeck’s Operation)

**Published:** 1869-02

**Authors:** 


Depression and Replacement of the Superior Maxilla (Langenbeck's Operation.) Service of Dr. Cheever. Case I.—*Naso-pharyngeal Polypus.*—(*Vide* Surgical Cases for 1867) The patient was a student, aged 19 years. Thirteen months previously to his entrance to the Hospital for his present disability, he had undergone an operation for the relief of disease of which this was the recurrence; and the history of the growth dated two years and a half before that time. Its development had given rise to the following symptoms: profuse epistaxis, complete obstruction of the nostril, and the discharge of a thin and offensive fluid. Examination discovered the presence of a firm, lobulated tumor pressing down on the soft palate and filling the upper and back part of the pharynx. The general condition of the patient was good, and the growth was not painful.

*Operation.*—It was removed by temporary depression of the right upper maxilla, as follows: The primary incision was carried from near the inner canthus of the eye, downward along the fold at the side of the nose, around the ala, and through the commissure of the upper lip. The flaps were reflected so as to expose the body of the bone. The symphysis of the jaw was divided along the hard palate, and a section made with a saw across the bone from the tuberosity into the middle meatus of the nose. The section of the bone was depressed, so that it was held only by its posterior attachments. The tumor was thus exposed and reached. Its attachments to the body of the sphenoid bone and to the upper and back part of the pharynx were divided with some difficulty with scissors, and the point of section (two inches square) cauterized with strong nitric acid. The bone was replaced and held well in position by a wire around the adjacent incisor teeth. The soft parts were easily apposed and retained by silk satures. The hæmorrhage was inconsiderable. The constitutional disturbance which followed the operation was comparatively slight. With a dressing of equal parts of tincture of myrrh and water, the wound healed satisfactorily, and throughout the convalescence there was no complication to impede recovery. In nineteen days the ligatures had all come away and there was no purulent discharge. The bone was in excellent position and motionless. After thirty-five days he was discharged well.

Symptoms of recurrence of the growth were noticed after eleven months. The nostril became obstructed as before, and there was a feeling of fullness in the head. Otherwise than this the tumor had caused no inconvenience. There was no appearance of any disease in the pharynx, but Dr. Langmaid, with the rhinoscope, discovered the fibrous mass occupying its former position and attached, like its predecessor, to the inferior aspect of the body of the sphenoid bone and to the adjacent region of the pharynx.

*Second Operation.*—The steps in the operation for its removal, the operation being performed at once, were almost identical with those of the former one, and the lines of section were in the cicatrices of those of the year previous. Owing, however, to the thickening of the bone in the course of healing, it was necessary to remove a small portion at the inner angle, just below the orbital process, in order to expose the growth. The tumor, which was of the size of an English walnut, was removed by section of its pedicle with scissors, and the bone was thoroughly scraped. The hæmorrhage was not sufficient to require ligatures. The bone and soft parts were opposed as in the former operation; a gutta-percha plug between the teeth and a bandage around the lower jaw, and over the head, aided in supporting the parts.

Convalescence, in this case, was more rapid, even, than in the primary operation. Without complication or drawback, recovery proceeded steadily, and after twenty-seven days he was discharged with the wounds perfectly healed, and the bone firmly in its place.

